# Comparison beetwen open and laparoscopic radical cistectomy in a latin american reference center: perioperative and oncological results

**DOI:** 10.1590/S1677-5538.IBJU.2014.0168

**Published:** 2015

**Authors:** Marcos Tobias-Machado, Danniel Frade Said, Anuar Ibrahim Mitre, Alexandre Pompeo, Antonio Carlos Lima Pompeo

**Affiliations:** 1 Departmento de Urologia, Seção de Urologia Oncológica da Faculdade de Medicina do ABC, Santo André, Brasil; 2 Departamento de Urologia - Faculdade de Medicina da Universidade de São Paulo

**Keywords:** Urinary Bladder, Cystectomy, Laparoscopy, Urinary Bladder Neoplasms

## Abstract

**Objectives:**

To evaluate the differences of peri-operatory and oncological outcomes between Laparoscopic Radical Cystectomy and Open Radical Cystectomy in our center.

**Materials and Methods:**

Overall, 50 patients were included in this non randomized match-pair analysis: 25 patients who had undergone Laparoscopic Radical Cystectomy for invasive bladder cancer (Group-1) and 25 patients with similar characteristics who had undergone Open Radical Cystectomy (Group-2). The patients were operated from January 2005 to December 2012 in a single Institution.

**Results:**

Mean operative time for groups 1 and 2 were 350 and 280 minutes (p=0.03) respectively. Mean blood loss was 330 mL for group 1 and 580 mL for group 2 (p=0.04). Intraoperative transfusion rate was 0% and 36% for groups 1 and 2 respectively (p=0.005). Perioperative complication rate was similar between groups. Mean time to oral intake was 2 days for group 1 and 3 days for group 2 (p=0.08). Median hospital stay was 7 days for group 1 and 13 for group 2 (p=0.04). There were no differences in positive surgical margins and overall survival, between groups.

**Conclusions:**

In a reference center with pelvic laparoscopic expertise, Laparoscopic Radical Cystectomy may be considered a safe procedure with similar complication rate of Open Radical Cystectomy. Laparoscopic Radical Cystectomy is more time consuming, with reduced bleeding and transfusion rate. Hospital stay seems to be shorter. Oncologically no difference was observed in our mid-term follow-up.

## INTRODUCTION

Surgery plays a major role in the treatment of all stages of invasive bladder cancer (1).

Open Radical Cystectomy (ORC) has been the gold standard technique. However, contemporary studies have shown that open radical cystectomy morbidity is higher than 50% in reference centers. Most significant complications, such as infections, paralytic ileus, operative wound dehiscence and urinary or intestinal fistulas can be life-threatening in about 10 to 20% of cases (2).

Laparoscopic Radical Cystectomy (LRC) was first performed in the 90s by Parra et al. (3) and the first laparoscopic radical cystectomy was reported by Sanchez de Badajoz et al. (4) in 1995. This minimally invasive technique seems to have a smaller complication rate and very similar oncologic parameters to classical open surgery (5–7). This procedure is gaining the interest of urologic oncologists (5). Some reports show that LRC compared to ORC has less blood loss and the patient has an early return to normal activities, reduction of postoperative pain and better cosmetic results. On the other hand, it is a procedure that requires minimally invasive surgery expertise, higher costs and a longer surgical time (5–7).

A preliminary report of Latin American multicenter experience suggested that LRC is feasible with acceptable complication rate (8). However LRC's potential advantages were not demonstrated because no comparative data to open surgery has been described.

We aimed to compare patients submitted to ORC (historical controls) and LRC performed in the same reference centers in Brazil matched for stage and clinical characteristics.

## MATERIALS AND METHODS

### 

#### Patients

After Institutional Review Board approval, from January 2005 to December 2012, a total of 50 patients diagnosed with invasive urothelial bladder tumor by endoscopic resection and an American Society of Anesthesiologists (ASA) score <3 underwent radical cystectomy with extended pelvic lymphadenectomy. We queried our database for patient demographics, preoperative disease characteristics, perioperative variables, and pathological outcomes. All patients had pre-operative staging by clinical, laboratorial and radiological exams, following the TNM staging system by the American Joint Committee on Cancer (1). The period between diagnosis and treatment of invasive urothelial cancer was less than 3 months in all cases. Bulky disease and patients with poor performance status were excluded from this comparative study.

The patients were paired by demographics and staging characteristics. No randomization was used and the patients were divided in two groups.

Group 1 was composed by 25 patients who underwent laparoscopic surgery (LRC) performed by an expert surgeon (MTM) which previous experience included 500 laparoscopic surgeries and of these 100 were laparoscopic radical prostatectomies. All cases included in this group represent the learning curve for LRC in our institution. Group 2 was formed by 25 patients that underwent open surgery (ORC) by three experienced uro-oncological surgeons from the same institution.

Perioperative and oncologic data were collected prospectively. Complications were classified according to Clavien-Dindo score.

#### Operative technique

The preoperative preparation included a mild laxative for colon cleaning and hospital stay prior the procedure with fasting for eight hours. Typing and blood reserve were routinely performed and all patients received antibiotic prophylaxis at the time of anesthesia induction. The procedure was performed under general anesthesia with endotracheal intubation and insertion of urethral catheter and nasogastric tube.

ORC was performed through a midline incision as previously described (9, 10).

On men, laparoscopic cystoprostatectomy was performed according to the technique described (5, 11). After the establishment of pneumoperitoneum with a Veress needle, one trocar 10/11mm was inserted 2cm above the umbilicus and a 0 degree lens was used for reviewing of the abdominal cavity. The other trocars were inserted under vision of the cavity. The trocars were arranged in an inverted V shape with one trocar at the apex for the lens, two other trocars of 10/12mm, and two final trocars of 5mm along the anterior-superior iliac spines (5).

**Figure 1:**
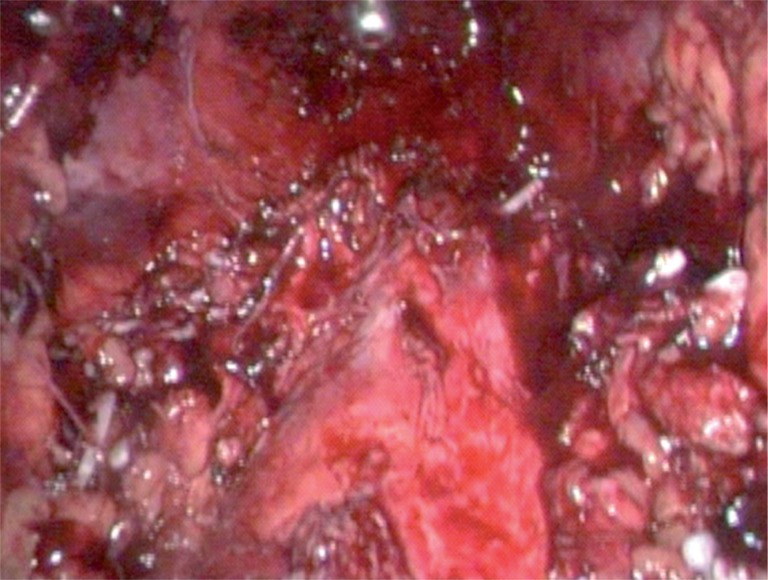
Pelvic cavity after specimen removal.

The specimen was suitably disposed in an endobag for subsequent removal through a periumbilical or Pfannestiel incision (5) of approximately 4-6cm and the pelvic cavity was reviewed after that (Figure-1).

**Figure 2:**
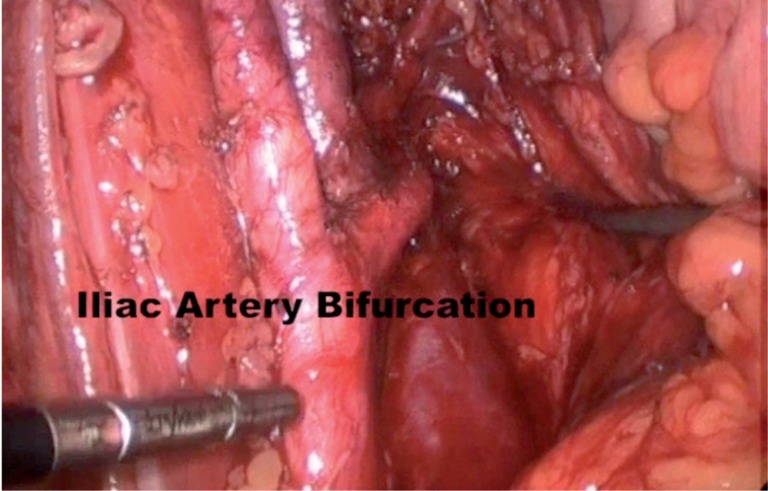
Major vessels after lymphadenectomy.

On women, the procedure was very similar; routinely we started with lymphadenectomy and the final surgical specimen including bladder, uterus and anterior portion of vagina were removed vaginally, and the dome of vagina was then sutured. Of note, the lymphadenectomy boundaries used during dissection included the genitofemoral nerve laterally, the bladder medially, Cloquet's node distally, the obturator nerve and its vessels posteriorly, and the mid-common iliac vessels proximally for both ORC and LRC (Figure-2).

We used three different types of urinary diversions according to the surgeon preference and the patient's condition: Studer orthotopic neobladder, Bricker ileal conduit and cutaneous ureterostomy. The ileal conduit is a popular technique of urinary diversion after radical cystectomy. Ileal neobladder reconstruction was performed only in selected patients with usable urethra. Cutaneous ureterostomy was performed in patients with advanced age and significant comorbidity (diabetes, anemia, COPD) (11). All urinary diversions were performed extra corporeally.

Postoperative follow-up was conducted at 3-month intervals during the first year, at 6-month intervals during the second year, and annually thereafter. Follow-up consisted of medical history, physical examination, and routine biochemical profile. Ultrasonography of the abdomen, urography, and chest X-rays were performed at 3, 6, and 12 months postoperatively, then annually unless otherwise clinically indicated. Abdominal/pelvic computed tomography scans were performed 6 months postoperatively and annually thereafter. The patients complications were cataloged during hospitalization and in clinical attendance for 90 days after surgery.

### Statistical analysis

Quantitative values were compared by Student's t test. Qualitative variables were compared by the chi-square test or Fisher's exact test.

## RESULTS

The mean age of patients was 65 years (range 55-81), the rate of male/female was 1:2, and the follow-up time was 47 months (range 10-58). The demographic and preoperative characteristics of both groups were similar and are presented in Table-1. There were no significant differences in gender, age, body mass index, ASA classification, number of previous abdominal surgeries. No patients received pelvic radiation or neoadjuvant chemotherapy.

**Table 1:** Demographic characteristics and perioperative data.

		LAPAROSCOPIC (group 1)	OPEN (group 2)	p Value
No. Patients		25	25	
Mean age±SD		63±10	65±8	0.9
Male/Female Rate		1:2	1:2	1.0
**TURB stage**				
	pT1	10	8	0.8
	pT2	15	17	
Mean BMI±SD		26.8±10	27.9±8	0.7
**ASA score, No. (%)**				
	1	1 (4)	2 (8)	0.8
	2	24 (96)	23 (92)	
Tabagism		20	19	0.8
Mean Operative Time (min) (range)		350 (240-400)	280 (200-350)	0.03
Mean Ablative Time (min) (range)		180 (125-200)	150 (120-185)	0.21
Mean Blood Loss (mL) (range)		330 (200-400)	580 (450-660)	0.04
Intraoperative Transfusion, No. (%)		0 (0)	9 (36)	0.005
Perioperative Complication, No. (%)		10 (40)	9 (36)	0.8
Mean Time to oral intake±SD (days)		2±0.9	3±1.1	0.08
Mean Hospital Stay±SD (days)		7±5.4	13±6.2	0.04

In Group 1 (LRC) the urinary diversions were: 10 (40%) neobladder; 13 (52%) Bricker; and 2 (8%) cutaneous ureterostomy. There were no conversions to open surgery. The mean operative time was 350 minutes, ranging between 240 and 400 minutes. The mean ablation time was 180 minutes, ranging between 125 and 200 minutes. The average blood loss of 330 mL (200-400 mL) was also estimated. No patient required blood transfusion in the intraoperative period. Participants in this group took two days to start oral diet and seven days to be discharged. None had to be reoperated.

In Group 2 (ORC) the urinary diversions were: 15 (60%) neobladder; and 10 (40%) Bricker. The mean operative time was 280 (200-350) minutes, mean ablation time was 150 (120-185) minutes. The estimated blood loss was 580 mL (450-660 mL), requiring intraoperative blood transfusion in 36% of patients (p=0.05). Unlike the patients in Group 1, the second group took three days to take oral diet and remained inpatients for 13 days after surgery. None of them required reoperation as well.

Perioperative complications were similar in both Groups. In Group 1 a total of 40% (10 patients) had complication: 20% Clavien 1, 12% Clavien 2 and 8% Clavien 3. In Group 2 a total of 36% (9 patients) had complications: 20% Clavien 1, 8% Clavien 2 and 8% Clavien 3. No Clavien 4-5 complications were observed in this study.

Perioperative pathological outcomes are showed in Table-2. In Group 1, there were 10 (40%) patients with preoperative staging pT1 and 15 (60%) pT2. In Group 2, 8 (32%) patients had preoperative stage pT1, 13 (52%) pT2 and four (16%) pT3. In the first group (LRC) there were 16±4 lymph nodes retrieved, 18% lymph node invasion. In the second group (ORC) there were 18±3 lymph nodes retrieved, 22% lymph node invasion. There were no statistical differences between the number of retrieved nodes. The median follow-up of the Groups was similar. There were also no differences in positive surgical margins (none in both groups) and overall survival presented similar oncological results.

**Table 2:** Postoperative histopathological results.

		LAPAROSCOPIC (group 1)	OPEN (group 2)	p Value
Nb tumor stage:				
	pT1	10	8	
	pT2	15	13	0.2
	pT3	0	4	
Mean lymph nodes retrieved±SD		16±4	18±3	0.6
**Lymph node involvement (%)**				
	pN0	82	78	0.5
	pN1 or grater	18	22	
Nb. positive surgical margins		0	0	1.0
Nb histology		25 urothelial bladder cancer	25 urothelial bladder cancer	1.0

## DISCUSSION

Expertise and reduction of complications to acceptable rates with radical cystectomy are achieved in high volume centers. Minimally invasive technique is acquiring interest from urologists in reference centers (5) and the benefits of this approach are being recognized. ORC is mainly adopted because it is less costly and there are few minimally invasive trained urologists for radical cystectomy in Brazil. In the U.S., since there are high volume centers with robotic expertise and available technology, this surgery is competing with open and laparoscopic cystectomy, and gaining ground in pelvic surgery (12).

We describe herein our experience with LRC and compare perioperative characteristics, oncological outcomes and complication rates with our ORC results. The findings suggest that LRC is associated with longer operating times, less blood loss, lower transfusion rates, and decreased hospital stays. Importantly, a significant decrease in morbidity rates with comparable short-term oncological outcomes was seen when comparing LRC with ORC.

There have been several published reports of LRC and ORC (Table-3). In those studies, laparoscopic radical cystectomy seems to have a lower morbidity rate than ORC (11, 13, 14).

**Table 3:** Comparison of perioperative characteristics.

	Porpiglia et al.		Hemal and Kolla		Guillotreau et al.		Present study	
	LRC	ORC	LRC	ORC	LRC	ORC	LRC	ORC
Operative time (min)	284	260	305	265	382.2	334.1	350	250
Ablative time (min)	-	-	107	101.3	161.1	181.7	180	150
Blood loss (mL)	520	770	414	825	429.7	923.2	330	580
Intraoperative transfusion	10%	18%	46.7%	80%	7.9%	36.7%	0%	36%
Perioperative complication	-	-	-	-	26.3%	60%	40%	36%
Time to oral intake (day)	3.3	5.7	3.7	5.1	3.8	6.4	2	3
Hospital stay (day)	18.1	19.7	9.2	11.8	12.7	15.6	7	13

Despite the longer operative time in LRC, our complication rate was not higher than ORC. Laparoscopic procedure also accelerate oral intake and reduced return to normal bowel function. Moreover, laparoscopy is also associated with a lower incidence of infectious complications due to shorter exposure of the abdominal cavity. According to Targarona et al., there is less involvement of the immune system in this procedure (11, 13, 14). Our results are similar to previous published reports. In our study, it was not observed a high rate of complications specific to the laparoscopic approach, such as leakage and fistulas. This may be due to our surgeon laparoscopic experience and to our urinary reconstruction performed extra corporeally, which is a safest way to decrease complications.

We also observed lower blood loss/transfusion, reduced hospital stay and similar mid-term oncological results. Lymph node dissection in invasive bladder tumors is important for both accurate staging and adequate oncological control. Our results showed that lymphadenectomy yield in LRC was not inferior to ORC. In previous laparoscopic studies (15, 16), the mean number of lymph nodes retrieved was similar to our study (1). Other retrospective comparative studies (14) also showed no statistically significant difference in lymph node yield between LRC and ORC demonstrating that minimally invasive procedure do not preclude an adequate node dissection. As LRC is a complex surgery the results could be improved with more experience.

Nevertheless, costs are an inherent discussion in any minimally invasive procedure. A recent study reported that LRC had lower costs compared to ORC (17.534 Euros compared to 22.284 Euros, respectively; p not significant). Authors emphasize that costs with disposable surgical equipment in LRC are compensated by lower transfusion rates, shorter hospital length and less intensive care admissions (17).

Our study has some obvious limitations. Both the patients and the surgeons could not be blinded because of the surgical nature of the trial. Our patient numbers were relatively small and the mean follow-up was relatively short. The lack of randomization prevented us to conclude which surgical procedure was better. Otherwise, the procedures were performed by a single laparoscopic surgeon and 3 open surgeons, but all with extensive experience regarding cystectomy. In relation to the selective criteria, our study demonstrated the initial feasibility, safety and oncological equivalence for cases with bladder disease <pT3 in patients with good performance status. Future studies are needed to evaluate safety in patients with poor performance status and oncologic efficacy for more advanced disease.

In conclusion, our study suggested that LRC is superior to ORC in terms of perioperative outcomes and may be used in experienced hands with the advantages of minimal invasive procedures. Despite the shorter follow-up and the small number of patients, our study demonstrated no major difference in oncological outcomes (12, 15, 16, 18). However, the limitations of our study do not allow a final conclusion and future large randomized controlled trials with longer follow-up are needed to provide more convincing oncological outcomes.
